# Chromosome-scale genome sequence assemblies of the ‘Autumn Bliss’ and ‘Malling Jewel’ cultivars of the highly heterozygous red raspberry (*Rubus idaeus* L.) derived from long-read Oxford Nanopore sequence data

**DOI:** 10.1371/journal.pone.0285756

**Published:** 2023-05-16

**Authors:** R. Jordan Price, Jahn Davik, Felicidad Fernandéz Fernandéz, Helen J. Bates, Samantha Lynn, Charlotte F. Nellist, Matteo Buti, Dag Røen, Nada Šurbanovski, Muath Alsheikh, Richard J. Harrison, Daniel James Sargent

**Affiliations:** 1 Cambridge Crop Research, NIAB, Cambridge, United Kingdom; 2 Division of Biotechnology and Plant Health, Norwegian Institute of Bioeconomy Research, Ås, Norway; 3 Department of Genetics, Genomics and Breeding, NIAB, East Malling, Kent, United Kingdom; 4 Department of Agriculture, Food, Environment and Forestry (DAGRI), University of Florence, Florence, Italy; 5 Graminor Breeding Ltd., Ridabu, Norway; Chiba Daigaku, JAPAN

## Abstract

Red raspberry (*Rubus idaeus* L.) is an economically valuable soft-fruit species with a relatively small (~300 Mb) but highly heterozygous diploid (2*n* = 2*x* = 14) genome. Chromosome-scale genome sequences are a vital tool in unravelling the genetic complexity controlling traits of interest in crop plants such as red raspberry, as well as for functional genomics, evolutionary studies, and pan-genomics diversity studies. In this study, we developed genome sequences of a primocane fruiting variety (‘Autumn Bliss’) and a floricane variety (‘Malling Jewel’). The use of long-read Oxford Nanopore Technologies sequencing data yielded long read lengths that permitted well resolved genome sequences for the two cultivars to be assembled. The *de novo* assemblies of ‘Malling Jewel’ and ‘Autumn Bliss’ contained 79 and 136 contigs respectively, and 263.0 Mb of the ‘Autumn Bliss’ and 265.5 Mb of the ‘Malling Jewel’ assembly could be anchored unambiguously to a previously published red raspberry genome sequence of the cultivar ‘Anitra’. Single copy ortholog analysis (BUSCO) revealed high levels of completeness in both genomes sequenced, with 97.4% of sequences identified in ‘Autumn Bliss’ and 97.7% in ‘Malling Jewel’. The density of repetitive sequence contained in the ‘Autumn Bliss’ and ‘Malling Jewel’ assemblies was significantly higher than in the previously published assembly and centromeric and telomeric regions were identified in both assemblies. A total of 42,823 protein coding regions were identified in the ‘Autumn Bliss’ assembly, whilst 43,027 were identified in the ‘Malling Jewel’ assembly. These chromosome-scale genome sequences represent an excellent genomics resource for red raspberry, particularly around the highly repetitive centromeric and telomeric regions of the genome that are less complete in the previously published ‘Anitra’ genome sequence.

## Introduction

Red raspberry (*Rubus idaeus* L.) is a popular, highly heterozygous diploid (2*n* = 2*x* = 14) perennial crop plant, with 822,493 tonnes of raspberries harvested annually throughout the world (http://www.fao.org/faostat/en/#data/QC). As such, red raspberry is one of the most economically valuable soft-fruit species, and interest in the development of new varieties through breeding and selection has led to many breeding programmes for the species being established globally. There are two main flowering habits in commercial red raspberry germplasm: floricane fruiting (such as the cultivar ‘Malling Jewel’) in which canes grown the previous season produce lateral shoots in the second year of growth that bear the flowers and fruits; and primocane fruiting (such as the cultivar ‘Autumn Bliss’), in which the first-year canes bear a limited number of flowers and fruits in the late summer or early autumn. Conventional breeding of red raspberry germplasm has led to many commercially-successful varieties being released, however genetic improvement in this highly heterozygous outbreeding species is slow [1] with several factors, including inbreeding depression, deleterious recessive alleles [2] and loss of fertility, amongst others, making the breeding process challenging. Plant genomics has the potential to significantly increase the precision and accuracy of breeding and selection of crop plants through the development and application of molecular markers, marker-assisted breeding and genomic selection. As such, significant molecular resources have been developed to date to support the breeding effort in red raspberry [3].

Chromosome-scale genome sequences are a vital tool in unravelling the genetic complexity controlling traits of interest in crop plants, as well as for functional genomics, evolutionary studies, and pan-genomics diversity studies. However, there are significant challenges in assembling genomes of highly heterozygous species using short-read sequence data because heterozygosity significantly increases the complexity of the de Bruijn graph structure predominantly used in short-read assemblers. Additionally, sequence length may make correct resolution of haplotigs in highly heterozygous genomes intractable, leading to fragmented assemblies containing many small contigs. Despite a relatively small haploid genome size of ~300 Mb, a diploid genome structure, and good progress in early sequencing efforts for red raspberry [4], the highly heterozygous nature of the genome, and the relatively high cost of early short-read sequencing data, meant that initial assemblies were highly fragmented and consisted of many thousands of scaffolds [5]. Thus, the development of chromosome-scale genomics resources for red raspberry has lagged behind those of closely-related species with genomes of a similar size such as *F*. *vesca* [6, 7], *Potentilla micrantha* [8] and *Rubus occidentalis* [9].

The advent of long-read sequencing technologies such as the platforms offered by Pacific Biosciences (PacBio) and Oxford Nanopore Technologies (ONT) has permitted high-quality, chromosome-scale genome sequence assemblies of many plant species, including those with relatively complex genomes such as the allo-octoploid strawberry *F*. *× ananassa* [7]. Long-read PacBio sequence data was recently used to construct chromosome-scale genome sequences of *R*. *idaeus* cultivars ‘Joan J’ [10] and ‘Anitra’ [11]. In the study of Davik *et al*., [11] the genome sequence covered 291.7 Mb, more than 99% of the estimated genome size of *R*. *idaeus*, with 85% of the sequence contigs resolved incorporated into seven chromosome-scale scaffolds, and 98% of Benchmarking Universal Single-Copy Orthologs (BUSCO) genes present in the assembly. The sequence of ‘Joan J’ [10] covered a total of ~300 Mb with a BUSCO completeness of 95.3%, but summary statistics for the genome were not available, and to date, the sequence from that publication has not been deposited in public sequence data repositories. These chromosome-scale genome sequences represent an excellent genomics resource for red raspberry, however additional sequence data and assemblies will help improve overall genome coverage and completeness, particularly around the highly repetitive centromeric and telomeric regions of the genome. More complete sequences will increase the value of these genomics resources for breeding and selection, as well as for more fundamental studies within the species.

Here we present the genome sequences for two red raspberry cultivars, ‘Autumn Bliss’ and ‘Malling Jewel’, along with associated gene predictions. ‘Malling Jewel’ [‘Preussen’ x (S1‘Lloyd George’ x S1‘Pyne’s Royal’ F2)] is a floricane red raspberry variety that was released in 1949 and represents a ‘pure’ *R*. *idaeus* genotype with 25% genetic contribution from *R*. *idaeus strigosus* (from ‘Superlative’) in a background of *R*. *idaeus vulgatus*. In contrast, ‘Autumn Bliss’, released in 1983 is a strict primocane variety with a complex hybrid genetic background [12] which has been used extensively as a parent in breeding worldwide for its early and productive primocane season, its aphid resistance, and its tolerance to soil-borne pathogens. The genome sequences produced in this investigation were compared to the recently-published genome sequence of the cultivar ‘Anitra’ [11]. The use of long-read ONT sequencing data yielded significantly longer read lengths than the PacBio sequence employed in the assembly presented by Davik *et al*. [11], which permitted more complete genome sequence assemblies to be resolved and in which the majority of the centromeric regions were defined.

## Materials and methods

### DNA extraction and sequencing

DNA was extracted from fresh, young leaf material collected from a single plant of the *R*. *idaeus* cultivars ‘Autumn Bliss’ and ‘Malling Jewel’ using a high molecular weight genomic DNA extraction protocol [13]. Long-read sequencing libraries were prepared using the SQK-LSK108 Ligation Sequencing Kit (Oxford Nanopore Technologies) from approximately 1 μg of high molecular weight genomic DNA, following the manufacturer’s protocol. Long-read libraries were sequenced on R9.4.1 Spot-On Flow cells (FLO-MIN106) using the GridION X5 platform (Oxford Nanopore Technologies) set to high accuracy base calling. A PCR free short read Illumina sequencing library was prepared for each of the two cultivars using an insert size of 450 bp. The ’Malling Jewel’ libraries were sequenced with 250 bp paired end reads on the Illumina HiSeq 2500 platform at the Earlham Institute (Norwich, UK) and the ‘Autumn Bliss’ libraries were sequenced with 300 bp paired end reads on the Illumina MiSeq platform at NIAB East Malling.

### RNA extraction and sequencing

Developing fruits of the red raspberry cultivar ‘Anitra’ were collected at three maturity stages; unripe, turning, and fully mature. Fruit samples from each maturity stage were collected at Graminor Njøs (Norway), where they were divided into three biological replicates, snap frozen in liquid nitrogen and stored at -80°C until the samples were processed further. Tissue samples were ground to a fine powder under liquid nitrogen, and total RNA was extracted using the RNeasy Plant Mini Kit (Qiagen, Germany) following the manufacturer’s instructions. The concentration and purity of the resultant RNA was measured using a QIAxpert spectrophotometer (Qiagen, Germany) and the integrity of the RNA was determined using a Qubit 4.0 fluorimeter (Thermo Fisher Scientific, UK). Samples with an RNA integrity number (RIN) value above 7.0 were submitted for subsequent RNA-Seq. Library preparations were performed using the NEB Next® ultra RNA Library Prep Kit (Biolabs, Inc., Beijing, China) and 150 bp paired-end sequencing was performed by Norwegian Sequencing Centre (Oslo University Hospital, Norway) using the HiSeq2500 platform (Illumina Inc., Beijing, China) to yield between 12.6 and 19.4 Gb of data per sample.

### Genome assembly

Before assembling the sequence data, the genome size of the ‘Autumn Bliss’ and ‘Malling Jewel’ genomes was estimated by counting k-mers (*n* = 27) in the Illumina reads in each dataset and calculating a histogram of the k-mer frequencies vs. counts using KAT [14], which were plotted using GenomeScope [15]. Long reads were quality controlled using NanoPlot v1.30.1 [16] and adapters were trimmed using Porechop v0.2.4 (https://github.com/rrwick/Porechop) using default parameters. Reads shorter than 1 Kb or with a quality score less than Q9 were removed using Filtlong v0.2.1 (https://github.com/rrwick/Filtlong). Long reads were assembled using NECAT v0.0.1_update20200803 [17] using a genome size of 300 Mb; all other parameters were left as default. Following assembly, heterozygous contigs and contig overlaps were identified and removed using Purge_Dups v1.0.1 [18] with default settings. Error correction was performed by aligning the long reads to the ‘Autumn Bliss’ and ‘Malling Jewel’ assemblies with Minimap2 v2.17-r941 [19] to inform one iteration of Racon v1.4.20 [20], followed by one iteration of Medaka v1.5.0 (https://github.com/nanoporetech/medaka) using the r941_min_high_g360 model. Illumina paired-end reads were quality controlled using FastQC v0.11.9 (https://www.bioinformatics.babraham.ac.uk/projects/fastqc/), and adapters and low-quality regions were trimmed using Trimmomatic v0.39 [21]. Short reads were aligned to the purged and corrected long-read assemblies using Bowtie2 v2.4.4 [22] and Samtools v1.12 [23] to allow for three iterations of polishing using Pilon v1.24 [24]. Reference-guided assembly was performed using RagTag v2.1.0 [25] with the *R*. *idaeus* cv. ‘Anitra’ chromosome-level assembly [11] as the reference. Assembly statistics were generated using a custom Python script, and single copy ortholog analysis was performed using BUSCO v5.2.2 [26], using the eudicots_odb10 database.

### Gene prediction and annotation

Repetitive and low complexity sequences in the reference-guided assemblies for ‘Autumn Bliss’ and ‘Malling Jewel’ were identified and soft masked using Red version: 05/22/2015 [27]. The *R*. *idaeus* cv. ‘Anitra’ RNA-seq reads were quality controlled using FastQC v0.11.9 (https://www.bioinformatics.babraham.ac.uk/projects/fastqc/), and adapters and low-quality regions were trimmed using Trimmomatic v0.39 [21]. Assemblies were indexed and RNA-seq reads were aligned using HISAT2 v2.2.1 [28] using default settings. Gene prediction was performed using BRAKER2 v2.1.6 [29] under the–etpmode setting with the RNA-seq and the eudicots protein database as evidence. Annotation completeness of the genome was assessed using BUSCO v5.2.2 [26], using default parameters and the gene families set defined for the eudicots_odb10 database. Gene predictions for the purged alternative contigs in each assembly were performed and gene predictions unique to the these contigs were identified using blastn. The repeat co-ordinates file output from Red and the ‘gene’ co-ordinates from the annotations file output from BRAKER2 were plotted using pyCircos (https://github.com/ponnhide/pyCircos) to visualise the distribution and density of repeat and coding regions.

### Tandem repeat content

Tandem Repeats Finder [30] was used to analyse the tandem repeat regions in the assemblies of ‘Autumn Bliss’, ‘Malling Jewel’ and ‘Anitra’ using the following settings; match = 2, mismatch = 7, delta = 7, match probability = 80, indel probability = 10, minimum alignment score = 50, max period = 2000.

### Functional annotation

Pairwise sequence comparison of the predicted proteomes of each genome, along with the genes found uniquely in the alternative purged contigs was performed using the BLAST+ blastp-fast algorithm [31] through the Galaxy platform [32] against the NCBI nr, SwissProt, RefSeq, TrEMBL and Araport11 protein databases using an expectation value cutoff of 1e-6. InterProScan v5 [33] was used to assign InterPro domains and Gene Ontology (GO) terms, whilst BlastKOALA v2.2 [34] and eggNOG-mapper v2 [35] were used to map Kyoto Encyclopaedia of Genes and Genomes (KEGG) orthologs and KEGG pathways respectively. GO enrichment analyses were carried out using Cytoscape 3.9.1 [36] and the BinGO 3.0.3 plug-in [37] performing a hypergeometric test with False Discovery Rate (FDR) correction, a 0.05 significance level and GO Full ontology.

### Comparative genomics

Syntenic alignments to the ‘Anitra’ genome sequence [11] were generated and plotted using D-genies [38] implementing Minimap2 v2.17-r941 [19] for the main assemblies for both ‘Autumn Bliss’ and ‘Malling Jewel’. Additionally, the alternative contigs purged from heterozygous regions of the genome during assembly were plotted against the primary assemblies of each cultivar to determine their genomic positions. OrthoFinder [39] was used to identify gene families in the ‘Autumn Bliss’, ‘Malling Jewel’ and ‘Anitra’ genome sequences [11], along with the genome sequences of the related species *R*. *chingii* v1.0 [40], *R*. *occidentalis* v3.0 [41] and *Fragaria vesca* v4.0.a2 [42]. Presence or absence of genes in orthogroups was used to determine species- and cultivar-specific gene families.

## Results

### Long- and short-read sequencing data

Sequencing of high molecular weight genomic DNA from ‘Autumn Bliss’ and ‘Malling Jewel’ using long read ONT libraries yielded 15.8 Gb and 12.0 Gb of data for the two cultivars respectively. Following filtering, the sequencing datasets contained 833,623 reads for ‘Malling Jewel’ and 991,952 reads for ‘Autumn Bliss’, representing 55× and 42× coverage, respectively. The read length N_50_ was 37.5 Kb for ‘Malling Jewel’ and 18.4 Kb for ‘Autumn Bliss’, with maximum read lengths of 456,177 bp and 366,503 bp, respectively for the two cultivars (Table 1). After adapter trimming and the removal of low-quality sequence data, a total of 18.7 Gb of 250 bp paired-end Illumina sequencing data was produced for ‘Malling Jewel’ and 5.0 Gb of 300 bp paired-end Illumina sequencing data was produced for ‘Autumn Bliss’, representing 69× and 19× genome coverage, respectively.

**Table 1:** Filtered Oxford Nanopore reads used for the ‘Malling Jewel’ and ‘Autumn Bliss’ genome sequence assemblies.

	‘Autumn Bliss’	‘Malling Jewel’
Mean read length (bp)	11,484	18,004
Mean read quality	13.4	13.2
Number of reads	991,952	833,623
Read length N_50_	18,356	37,581
Total bases	11,391,903,736	15,008,949,175
Longest read (bp)	366,503	456,177

### Genome sequence assembly

The k-mer analysis performed for ‘Autumn Bliss’ returned a predicted heterozygosity of 1.54% and a total predicted genome length of 196.8–198.5 Mbp. The data for ‘Malling Jewel’ returned a predicted heterozygosity of 0.45% and a total predicted genome length of 294.9–298.6 Mbp (S1 Fig). Following long-read assembly, removal of heterozygous contigs and polishing using long- and short- read sequence data, the resulting assemblies for ‘Autumn Bliss’ and ‘Malling Jewel’ were 268.7 Mb and 265.7 Mb in length, and were composed of 146 and 86 contigs, respectively. The ‘Malling Jewel’ assembly had an N_50_ of 9.9 Mb with an L_50_ of seven contigs, whilst the ‘Autumn Bliss’ assembly had an N_50_ of 3.3 Mb and an L_50_ of 25 contigs (Table 2). A total of 339 contigs in ‘Autumn Bliss’ and 154 contigs in ‘Malling Jewel’ were purged from the heterozygous regions in the sequence assemblies. The total length of the purged contigs was 202.9 Mb and 235.6 Mb ‘Autumn Bliss’ and ‘Malling Jewel’ respectively. BUSCO analysis revealed 97.7% complete single copy orthologs in ‘Autumn Bliss’ and 97.6% in ‘Malling Jewel’, indicating that both assemblies contained high levels of gene space completeness (Table 2).

**Table 2:** Assembly statistics for the *de novo* assemblies of the *Rubus idaeus* ‘Malling Jewel’ and ‘Autumn Bliss’ genome sequences.

	‘Autumn Bliss’	‘Malling Jewel’
Total sequence length (bp)	268,668,490	265,724,784
Number of contigs	146	83
Longest contig (bp)	9,660,097	40,892,532
Shortest contig (bp)	3,465	3,226
GC content (%)	37.8	37.85
Contig N_50_ (bp)	3,253,875	9,899,270
Contig L_50_	25	7
Gap (%)	0	0
Complete BUSCOs (%)	97.7	97.6
Single copy BUSCOs (%)	89	93.6
Duplicated BUSCOs (%)	8.7	4
Fragmented BUSCOs (%)	0.4	0.4
Missing BUSCOs (%)	1.8	2

### Genome sequence scaffolding

The *de novo* assemblies of ‘Autumn Bliss’ and ‘Malling Jewel’ were scaffolded against the seven pseudochromosomes of the ‘Anitra’ genome sequence. Following scaffolding, 136 contigs of ‘Autumn Bliss’ and 79 contigs of ‘Malling Jewel’ were anchored to the seven ‘Anitra’ pseudochromosomes (Table 3). In total, 263.0 Mb (97.9%) of the ‘Autumn Bliss’ assembly was anchored to the ‘Anitra’ chromosomes, with a largest scaffold of 50.7 Mb and an N_50_ of 35.7 Mb, whilst 265.5 Mb (99.9%) of the ‘Malling Jewel’ assembly was anchored to the ‘Anitra’ chromosomes, with a largest scaffold of 44.5 Mb and an N_50_ of 36.1 Mb (Table 4). Of the ten contigs that were not anchored from the ‘Autumn Bliss’ assembly, nine were included in other contigs, whilst the longest contig (4.6 Mb) contained highly repetitive sequences. The four contigs from the ‘Malling Jewel’ assembly that were not anchored to the ‘Anitra’ chromosomes represented fragmented sequences of the chloroplast and mitochondrial genomes. The scaffolded, anchored assemblies were 15.5 Mb (6.3%) and 18 Mb (7.3%) larger than the previously published ‘Anitra’ genome sequence for ‘Autumn Bliss’ and ‘Malling Jewel’ respectively. Single copy ortholog analysis (BUSCO) revealed high levels of completeness in both genomes, with 97.4% of the sequences identified in ‘Autumn Bliss’ and 97.7% of the sequences identified in ‘Malling Jewel’ (Table 4). The seven pseudochromosomes assembled for each genome were numbered according to the ‘Anitra’ chromosomes which in turn are concordant with the chromosomes of other Rosoideae genomes such as *F*. *vesca* [6] and *Rosa chinensis* [43].

**Table 3:** *Rubus idaeus* ‘Malling Jewel’ and ‘Autumn Bliss’ contigs aligning to ‘Anitra’ genome pseudomolecules.

	‘Autumn Bliss’	‘Malling Jewel’
pseudochromosome 1	16	9
pseudochromosome 2	19	13
pseudochromosome 3	23	5
pseudochromosome 4	12	15
pseudochromosome 5	19	10
pseudochromosome 6	30	15
pseudochromosome 7	17	12
Total	136	79

**Table 4:** Assembly statistics for the reference scaffolded assemblies of the *Rubus idaeus* ‘Anitra’, ‘Malling Jewel’ and ‘Autumn Bliss’ genome sequences.

	‘Anitra’	‘Autumn Bliss’	‘Malling Jewel’
Total sequence length (bp)	247,480,545	262,950,453	265,504,618
Number of scaffolds	7	7	7
Longest scaffold (bp)	43,293,752	50,701,465	44,467,746
Shortest scaffold (bp)	28,685,549	32,095,515	34,604,543
GC content (%)	37.62	37.79	37.84
Scaffold N_50_ (bp)	34,491,998	35,733,366	36,052,904
Scaffold L_50_	4	4	4
Gap (%)	0.1	0	0
Masked sequence (%)	31.7	32.6	34.8
Complete BUSCOs (%)	97.4	97.4	97.6
Single Copy BUSCOs (%)	93.3	90.1	93.9
Duplicated BUSCOs (%)	4.1	7.3	3.8
Fragmented BUSCOs (%)	0.5	0.3	0.3
Missing BUSCOs (%)	2.1	2.3	2
Contigs aligned to ‘Anitra’ reference		136	79
Sequence aligned to ‘Anitra’ reference (%)		97.9	99.9

All raw sequencing data and genome assemblies presented here are available at the NCBI under the Bioproject IDs PRJNA886864, PRJNA886865 and PRJNA886875. The assemblies of the ‘Autumn Bliss’ and ‘Malling Jewel’ genomes, along with the transcriptome annotations of the ‘Autumn Bliss’, ‘Malling Jewel’ and ‘Anitra’ have also been deposited on the Genome Database for Rosaceae [44].

### Assembly completeness

**Fig 1:**
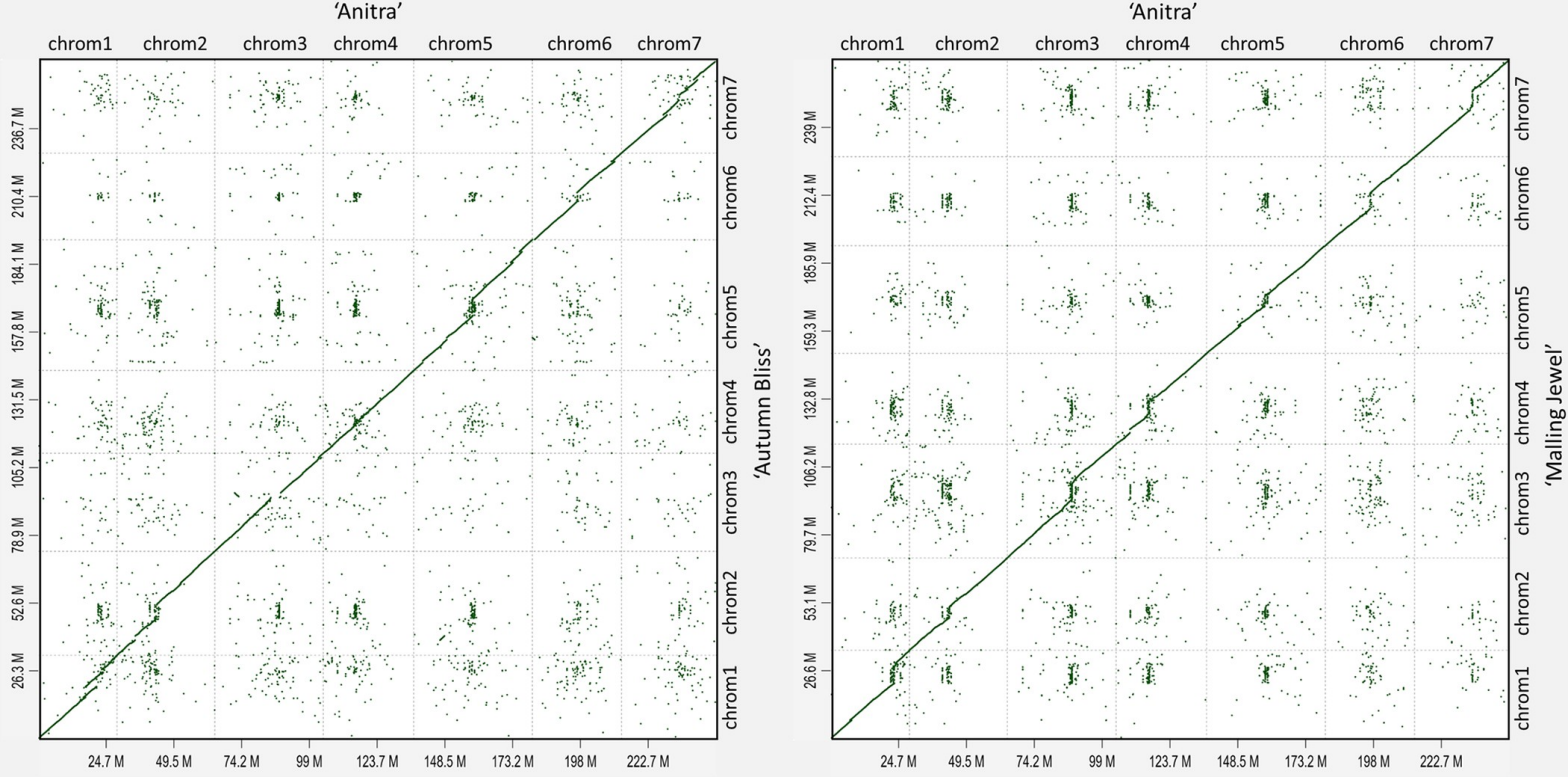
The red raspberry genome assemblies of ‘Autumn Bliss’ and ‘Malling Jewel’ show synteny to the previously published ‘Anitra’ genome. The dot plots show the alignments of ‘Autumn Bliss’ and ‘Malling Jewel’ genome assemblies to the ‘Anitra’ genome sequence. The plots show the synteny between ‘Autumn Bliss’ and ‘Malling Jewel’ contigs (*y*-axis) aligning to the ‘Anitra’ pseudochromosomes (*x*-axis). ‘Anitra’ chromosome names are given along with total assembly lengths of the ‘Anitra’, ‘Autumn Bliss (a) and ‘Malling Jewel’ (b) chromosomes in Mbp. The hashed horizontal lines indicate the positions along the *y*-axis of the assembled scaffolds in each assembly. ‘Autumn Bliss’ and ‘Malling Jewel’ scaffold names and sizes are given in S2 Fig.

**Fig 2:**
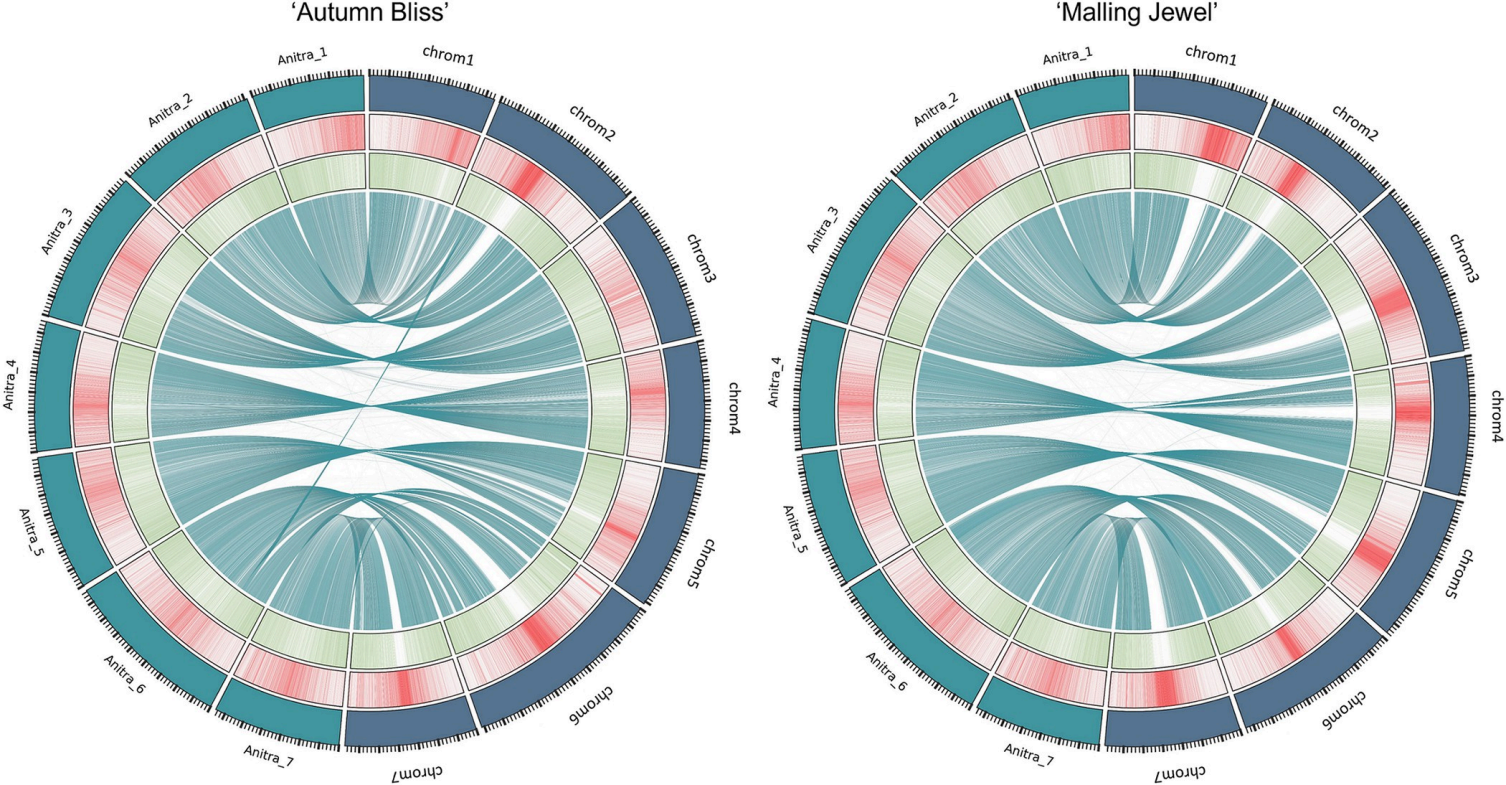
Extensive repetitive regions were identified throughout the pseudochromosomes of the ‘Anitra’, ‘Autumn Bliss’ and ‘Malling Jewel’ genome sequence assemblies. Circos plots showing the distribution of coding regions (green) and repetitive regions (red) in the ‘Autumn Bliss’, ‘Malling Jewel’ and ‘Anitra’ genome sequence assemblies along with the alignment of syntenic regions of ‘Autumn Bliss’ and ‘Malling Jewel’ to the ‘Anitra’ genome. Major tick marks indicate 5 Mb intervals and minor ticks indicate 1 Mb intervals.

**Fig 3:**
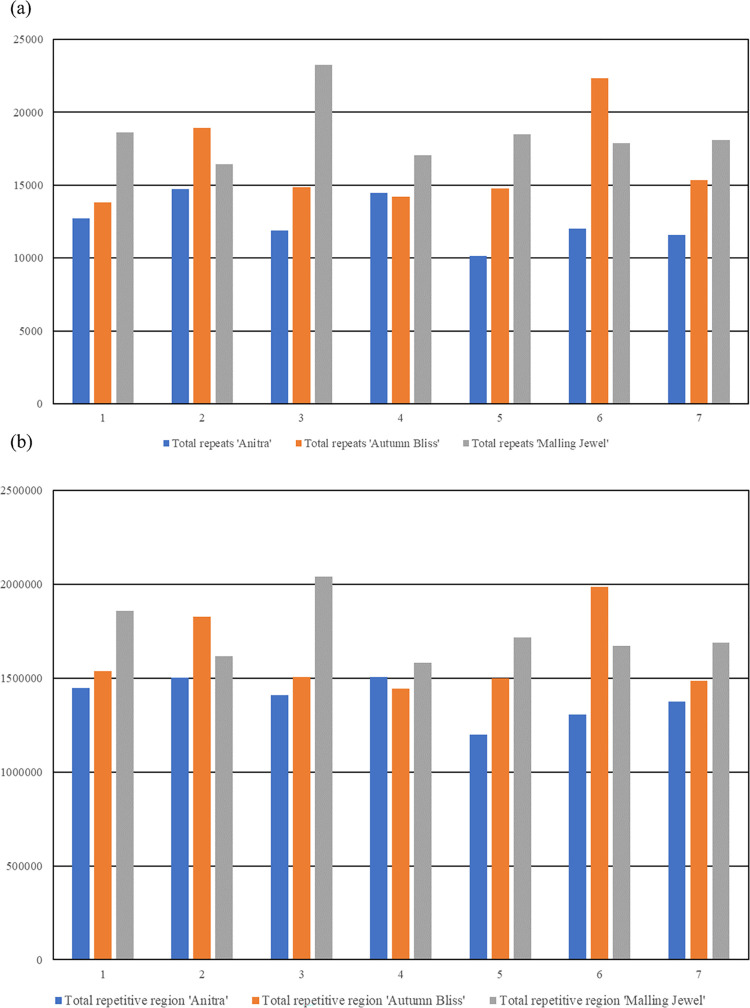
The density of repetitive sequences in the ‘Autumn Bliss’ and ‘Malling Jewel’ genome assemblies was substantially higher than in the previously published ‘Anitra’ assembly. Bar charts displaying the number of tandem repeat regions and total repetitive regions identified per chromosome in the ‘Anitra’, ‘Autumn Bliss’ and ‘Malling Jewel’ genomes using Tandem Repeat Finder.

The assembled ‘Autumn Bliss’ and ‘Malling Jewel’ contigs were highly syntenic and concordant with the previously published *R*. *idaeus* cv. ‘Anitra’ genome sequence of Davik et al. [11], despite being significantly larger than the ‘Anitra’ pseudochromosome assembly (Fig 1). S2 Fig contains the names and positions of the ‘Autumn Bliss’ and ‘Malling Jewel’ scaffolds along the ‘Anitra’ assembly, along with their lengths in base pairs. S1 File shows the relative positions of the alternative contigs purged from the primary assembly from heterozygous regions of the genome in relation to the associated primary assembly of each cultivar. The additional length of the primary assemblies presented was partly due to 15% of the total ‘Anitra’ assembly length not being scaffolded into the seven main pseudochromosomes, however there were also differences in the abundance of repetitive sequence within the genomes. Extensive repetitive regions were identified throughout the pseudochromosomes of all three assemblies (Fig 2) and the relative abundance of sequence within highly repetitive regions in the assemblies was compared to assess genome completeness. Regions with a higher density of repetitive sequence and lower gene density were observed near the centre of each of the seven ‘Anitra’ pseudomolecules, which we inferred correspond to the centromeres of each the seven chromosomes. These same regions were clearly identifiable in the ‘Autumn Bliss’ and ‘Malling Jewel’ pseudomolecules, and the density of repetitive sequence they contained was significantly higher than in the ‘Anitra’ assembly (Fig 3). The ‘Autumn Bliss’ and ‘Malling Jewel’ assemblies contained a greater total repetitive sequence within the centromeric regions, and more tandem repeat sequences overall on all seven pseudochromosomes than the ‘Anitra’ assembly, except for chromosome 4 of ‘Autumn Bliss’ (Fig 3). Additionally, telomeric repeat sequences ([CCCTAAA]n) were identified at both ends of five pseudochromosomes and at one end of the remaining two pseudochromosomes in ‘Malling Jewel’, whilst one pseudochromosome of the ‘Autumn Bliss’ assembly contained identifiable telomeric repeats at both ends, and a further three contained identifiable repeats at a single end.

### Gene prediction and annotation

A total of 41,800 protein coding regions were identified in the ‘Autumn Bliss’ assembly, whilst 40,491 were identified in the ‘Malling Jewel’ assembly. The distribution of these protein coding regions across the seven pseudochromosomes for each cultivar is shown in Fig 2. A further 1,023 genes in ‘Autumn Bliss’ and 1,227 genes in ‘Malling Jewel’ not present in the primary assemblies were found in the purged contigs. A BUSCO analysis of the proteome of the two assemblies identified 96.9% queried proteins in the ‘Autumn Bliss’ proteome, and 97.7% in the ‘Malling Jewel’ proteome (Table 5). The ‘Anitra’ genome sequence was re-annotated using the same methodology to ensure consistency in downstream orthology analyses [45]. The re-annotation of the ‘Anitra’ assembly resulted in 41,509 protein coding regions, with a BUSCO completeness score of 97.3% compared to 39,448 (91.3%) in the previous study of [11] indicating a largely complete gene-space characterisation in all three genomes. The statistics for the number of protein-coding gene predictions for each cultivar that returned ≥1 positive hits after the BlastP analysis with nr, Araport11, RefSeq, SwissProt and TrEMBL databases as subjects, are given in Table 6, along with the number of protein-coding gene regions assigned Interpro, GO, KEGG orthology and KEGG pathway terms. Best matches resulting from BlastP homology searches for each dataset are detailed in S2 File Gene prediction BlastP summary, whilst the functional annotation results are given in S3 File Gene prediction functional annotation.

**Table 5:** A comparison of Benchmarking Universal Single-Copy Orthologs (BUSCO) analysis of the proteome from the previously published ‘Anitra’ genome, the reannotated ‘Anitra’ genome, ‘Malling Jewel’ and ‘Autumn Bliss’.

	‘Anitra’ (Published)	‘Anitra’ (This study)	‘Autumn Bliss’	‘Malling Jewel’
Proteins	39,448	41,509	42,823	41,718
Complete (%)	91.3	97.3	96.9	97.7
Single (%)	84	83.2	83.4	87
Duplicated (%)	7.3	14.1	13.5	10.7
Fragmented (%)	3.7	0.9	0.9	0.6
Missing (%)	5	1.9	2.1	1.7

**Table 6:** Summary statistics for the number of protein-coding gene predictions for ‘Anitra’, ‘Autumn Bliss’ and ‘Malling Jewel’ that returned ≥1 positive hit after the BlastP analysis with nr, Araport11, RefSeq, SwissProt and TrEMBL databases as subjects, along with the number of protein-coding gene regions assigned Interpro, GO, KEGG orthology and KEGG pathway terms.

Cultivar	‘Anitra’	‘Autumn Bliss’	‘Malling Jewel’
Homology			
Predicted proteins #	41,509	42,823	41,718
BlastP vs NCBI nr: hits #	36,863	37,195	35,983
BlastP vs NCBI: hits nr %	88.60%	86.86%	86.25%
BlastP vs Araport11: hits #	32,326	32,271	31,129
BlastP vs Araport11: hits %	77.90%	75.36%	74.62%
BlastP vs RefSeq: hits #	36,778	37,100	35,878
BlastP vs RefSeq: hits %	88.60%	86.64%	86%
BlastP vs SwissProt: hits #	28,587	28,641	27,700
BlastP vs Swissprot: hits %	68.90%	66.88%	66.40%
BlastP vs Trembl: hits #	36,245	36,563	35,368
BlastP vs Trembl: hits %	87.30%	85%	84.78%
Functional Annotation			
InterPro: total hits #	95,663	93,586	90,763
InterPro: proteins with hits #	31,379	30,991	29,790
InterPro: proteins with hits %	75.60%	74.10%	73.60%
GO: total hits #	52,755	71,290	49,974
GO: proteins with hits #	22,732	22,223	21,554
GO: proteins with hits %	54.80%	53.20%	53.20%
KEGG ortholog: total hits #	11,363	11,212	10,780
KEGG ortholog: proteins with hits #	11,359	11,208	10,776
KEGG ortholog: proteins with hits %	27.40%	26.80%	26.60%
KEGG pathway: total hits #	36,760	36,726	34,649
KEGG pathway: proteins with hits #	9,190	9,152	8,732
KEGG pathway: proteins with hits %	0.221397769	0.218947368	0.215652861

### Orthology analysis

**Fig 4:**
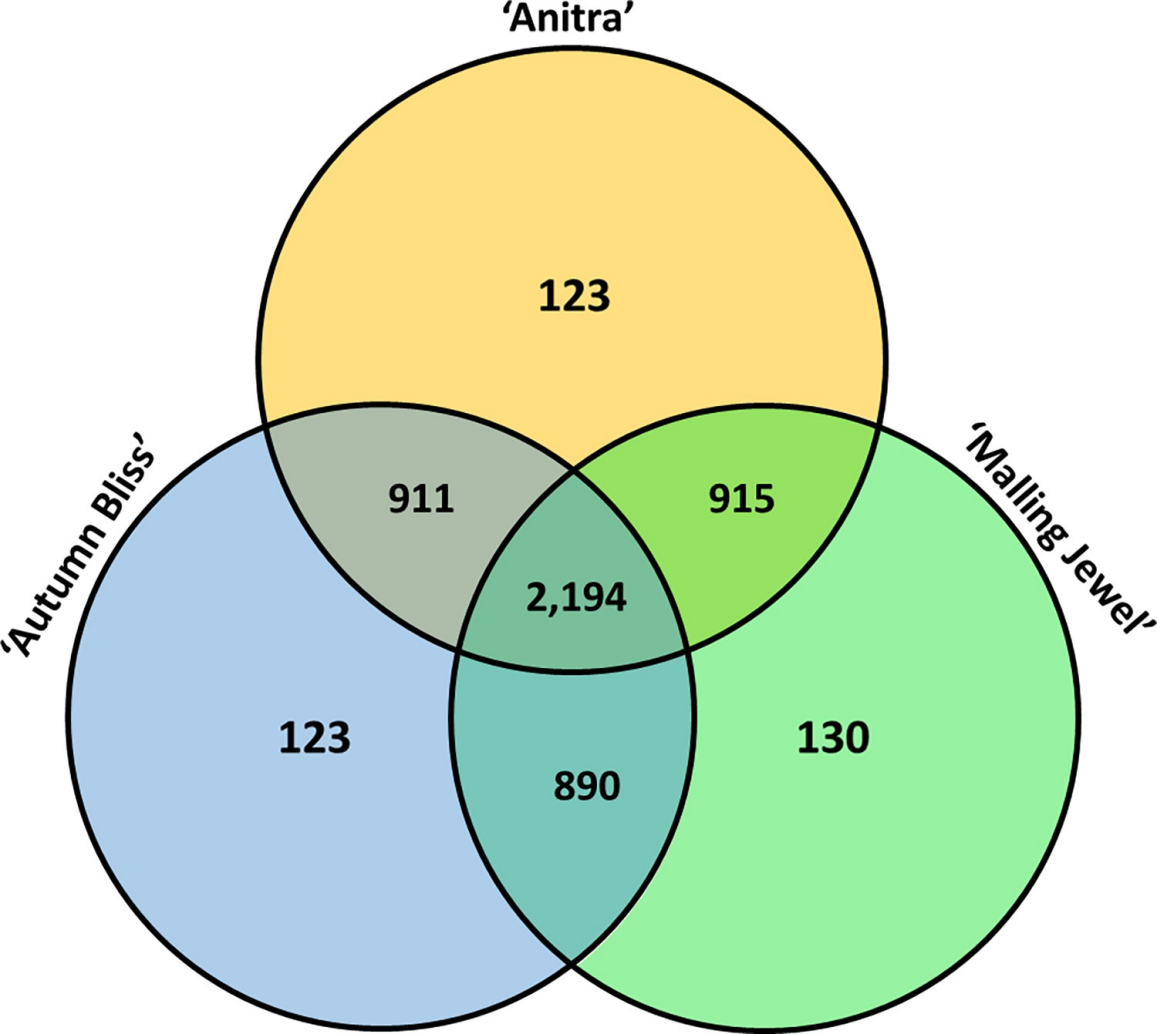
A Venn diagram showing the distribution of the 5,286 *Rubus idaeus*-specific orthogroups identified in this study between the ‘Anitra’, ‘Autumn Bliss’ and ‘Malling Jewel’ genomes.

**Fig 5:**
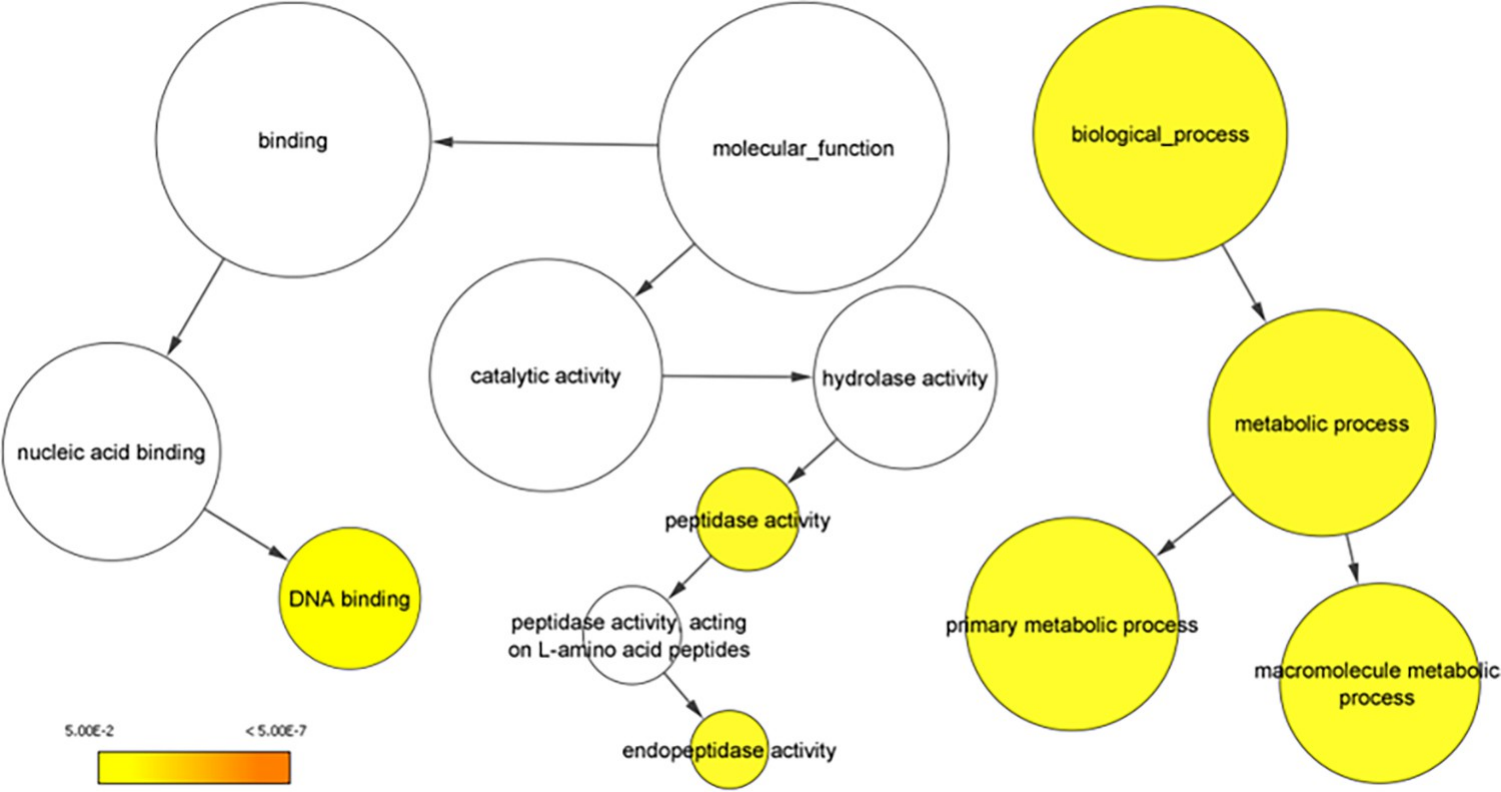
Overrepresented Gene Ontology (GO) categories in the orthogroups shared across ‘Anitra’, ‘Autumn Bliss’ and ‘Malling Jewel’ cultivars highlight key conserved processes. The circles are shaded based on significance level (yellow = False Discovery Rate (FDR) below 5.00E-2), and the radius of circles is proportional to the number of orthogroups included in each GO category.

A total of 34,213 orthogroups were identified in the comparison between the proteomes of the three *R*. *idaeus* cultivars, *R*. *occidentalis* [v3,9], *R*. *chingii* [v1, 40] and *F*. *vesca* [v4.0.a2, 42]. Of the identified orthogroups, 15,412 were shared across all species, whilst 5,286 orthogroups, containing 17,233 genes, were specific to *R*. *idaeus*. Of the *R*. *idaeus* specific orthogroups, 2,194 were shared across all three cultivars. Of the remaining groups, 915 were shared between ‘Anitra’ and ‘Malling Jewel’, 911 were shared between ‘Anitra’ and ‘Autumn Bliss’, and 890 were shared between ‘Malling Jewel’ and ‘Autumn Bliss’. Finally, 123 orthogroups were specific to ‘Anitra’, 130 to ‘Malling Jewel’ and 123 ‘Autumn Bliss’, representing 322, 303 and 305 genes, respectively (Fig 4). GO enrichment analysis of the seven *R*. *idaeus* subgroups using all the orthogroups as background showed that the orthogroups shared across the three cultivars ‘Malling Jewel’, ‘Anitra’ and ‘Autumn Bliss’ were significantly enriched for metabolic process (GO:0008152), primary metabolic process (GO:0044238), macromolecule metabolic process (GO:0043170), DNA binding (GO:0003677), peptidase activity (GO:0008233) and endopeptidase activity (GO:0004175) GO categories (Fig 5). Orthogroups shared between ‘Anitra’ and ‘Malling Jewel’, between ‘Anitra’ and ‘Autumn Bliss’, between ‘Malling Jewel’ and ‘Autumn Bliss’, as well as orthogroups unique to ‘Malling Jewel’, ‘Autumn Bliss’ and ‘Anitra’, were not significantly enriched for any GO category.

## Discussion

Plant genomes are often highly heterozygous and are composed of a significant proportion of highly repetitive DNA [46]. As such, large and highly contiguous assemblies are often intractable with short-read sequence data alone. In this report, we present two new chromosome-scale genome sequence assemblies for red raspberry (*R*. *idaeus*) derived from the cultivars ‘Autumn Bliss’ and ‘Malling Jewel’ which were constructed using long-read ONT sequence data and were compared to the previously published genome sequence assembly of the *R*. *idaeus* cultivar ‘Anitra’ [11].

Prior to scaffolding with long-range structural information, both genomes sequenced in this investigation returned a high level of contiguity, with just 143 and 83 contigs and a contig L_50_ of 25 and 7 returned for the ‘Autumn Bliss’ and ‘Malling Jewel’ assemblies respectively. This is in contrast to the 2,350 contigs in the ‘Anitra’ assembly, with both new assemblies containing at least 15 Mb more scaffolded sequence data than the ‘Anitra’ sequence [11]. The lower degree of contiguity within the ‘Autumn Bliss’ assembly is likely due to the differences in the long-read N_50_ values (37.5 Kb vs 18.4 Kb in ‘Malling Jewel’ and ‘Autumn Bliss’) of the sequence data returned for each cultivar. In contrast to the ONT data used in this investigation, Davik et al. [11] used PacBio long-read sequence data for the chromosome-scale assembly of the ‘Anitra’ genome. The PacBio reads used in that assembly had a mean sub-read length of 9.5 Kb and an N_50_ length of 13.6 Kb, which is significantly shorter than the N_50_ read length of the ‘Autumn Bliss’ and ‘Malling Jewel’ datasets presented here. In the final assembly, the highly repetitive centromeric regions of the ‘Anitra’ assembly were less well resolved than the gene-rich regions of the genome and significantly less well resolved than the same regions in the ‘Autumn Bliss’ and ‘Malling Jewel’ genomes presented here. The greater fragmentation in the pre-scaffolded ‘Anitra’ contigs than in the ‘Autumn Bliss’ and ‘Malling Jewel’ assemblies resulted in a significantly shorter final scaffolded sequence for ‘Anitra’, demonstrating the value of very long-read sequence data in resolving highly heterozygous genome sequences. The two new genome assemblies for red raspberry presented here displayed a very high degree of synteny with the genome sequence of ‘Anitra’. Analysis of the tandem repeats in the genome sequences of ‘Autumn Bliss’, ‘Malling Jewel’ and ‘Anitra’ demonstrated that much of the additional sequence data incorporated into the new assemblies was contained in the centromeric regions, which were more accurately assembled in the ‘Autumn Bliss’ and ‘Malling Jewel’ genomes due to the longer read length of the ONT data used compared to the shorter PacBio data used in the ‘Anitra’ assembly.

Cultivated red raspberry varieties are derived from the interbreeding of both the European red raspberry (*R*. *idaeus* subsp. *idaeus* L.) and the North American red raspberry (*R*. *idaeus* subsp. *strigosus* L.) as well as other species and subspecies. However, the global genetic structure of red raspberry populations has yet to be extensively studied. Modern red raspberry varieties are derived from a relatively narrow genetic base [47], with just 20 clones accounting for the majority of the genetic diversity in 137 varieties of known pedigree that have been released worldwide between 1960 and 1992. However, *R*. *idaeus* is naturally an out-crossing species, and as such, high levels of genome differentiation and heterozygosity are a feature of red raspberry germplasm. The two varieties for which genome sequence assemblies are presented in this paper are from very different backgrounds within the red raspberry germplasm base. ‘Malling Jewel’ is a floricane with a relatively pure *R*. *idaeus* genetic background, containing several important founder clones in its pedigree, whilst ‘Autumn Bliss’ in contrast is a strict primocane variety, with a complex hybrid pedigree including contributions from *R*. *arcticus* and *R*. *occidentalis*, meaning these two genomes span a significant representative portion of cultivated red raspberry diversity.

Many traits that have been selected for during the domestication and breeding of red raspberry including large fruit size, flavour, colour and pest and disease resistance, and alleles controlling these traits are likely to have been through genetic bottlenecks, with not all favourable alleles passed to all breeding populations of the crop globally. The genes contained within the 5,286 *R*. *idaeus*-specific orthogroups identified in this study were enriched for metabolic process, DNA binding, peptidase activity and endopeptidase activity, however, there is likely to be further differentiation within the cultivated and wild red raspberry germplasm that exists globally. The genetic resources for the red raspberry cultivars ‘Autumn Bliss’ and ‘Malling Jewel’ presented here, along with the previously published sequence of the cultivar ‘Anitra’ are valuable resources to begin to understand the structure and function of the red raspberry pan-genome and provide a route to unlock the potential of red raspberry germplasm through precise identification and characterisation of genetic loci controlling traits of agronomic importance.

## Supporting information

S1 Fig Plots of the k-mer distribution determined from analysis of illumine reads generated for the (a) ‘Malling Jewel’ and (b) ‘Autumn Bliss’ genomes.
(PNG)

S2 Fig The names and positions of the ‘Autumn Bliss’ and ‘Malling Jewel’ scaffolds along the ‘Anitra’ assembly, along with their lengths in base pairs. (PNG)

S1 File The relative positions of the alternative contigs purged from the primary assembly from heterozygous regions of the (a) ‘Autumn Bliss’ and (b) ‘Malling Jewel’ genomes.
(XLSX)

S2 File Gene prediction BlastP summary. (XLSX)

S3 File Gene prediction functional annotation. (XLSX)
