# Supplementary figures and images for: Chromosome-scale genome sequence assemblies of the ‘Autumn Bliss’ and ‘Malling Jewel’ cultivars of the highly heterozygous red raspberry (Rubus idaeus L.) derived from long-read Oxford Nanopore sequence data
Source: PLoS One. 2023 May 16;18(5):e0285756. doi: 10.1371/journal.pone.0285756 (PMC10187893; doi:10.1371/journal.pone.0285756)

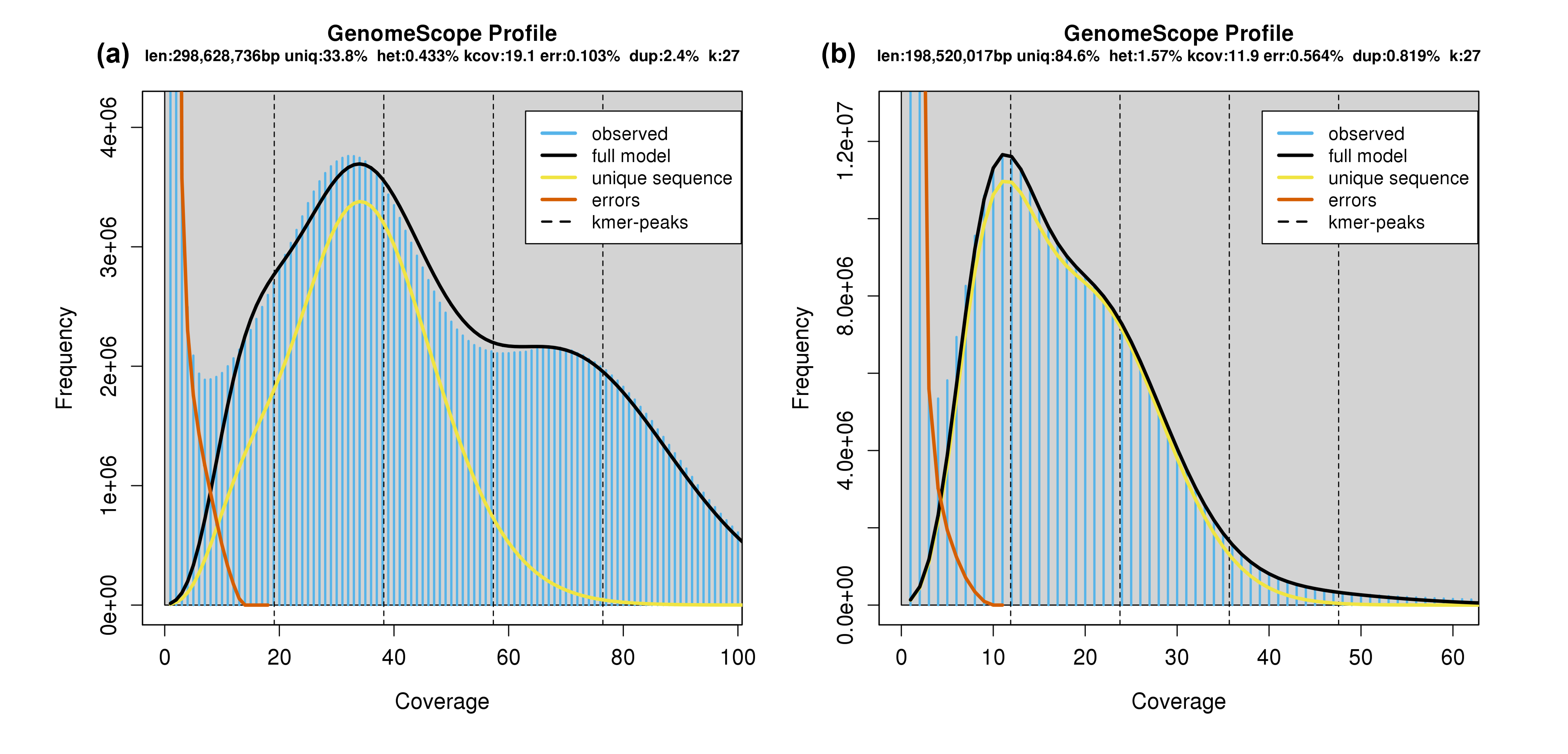

Supplement: S1 Fig — Plots of the k-mer distribution determined from analysis of illumine reads generated for the (a) ‘Malling Jewel’ and (b) ‘Autumn Bliss’ genomes. (PNG) [file pone.0285756.s001.png]

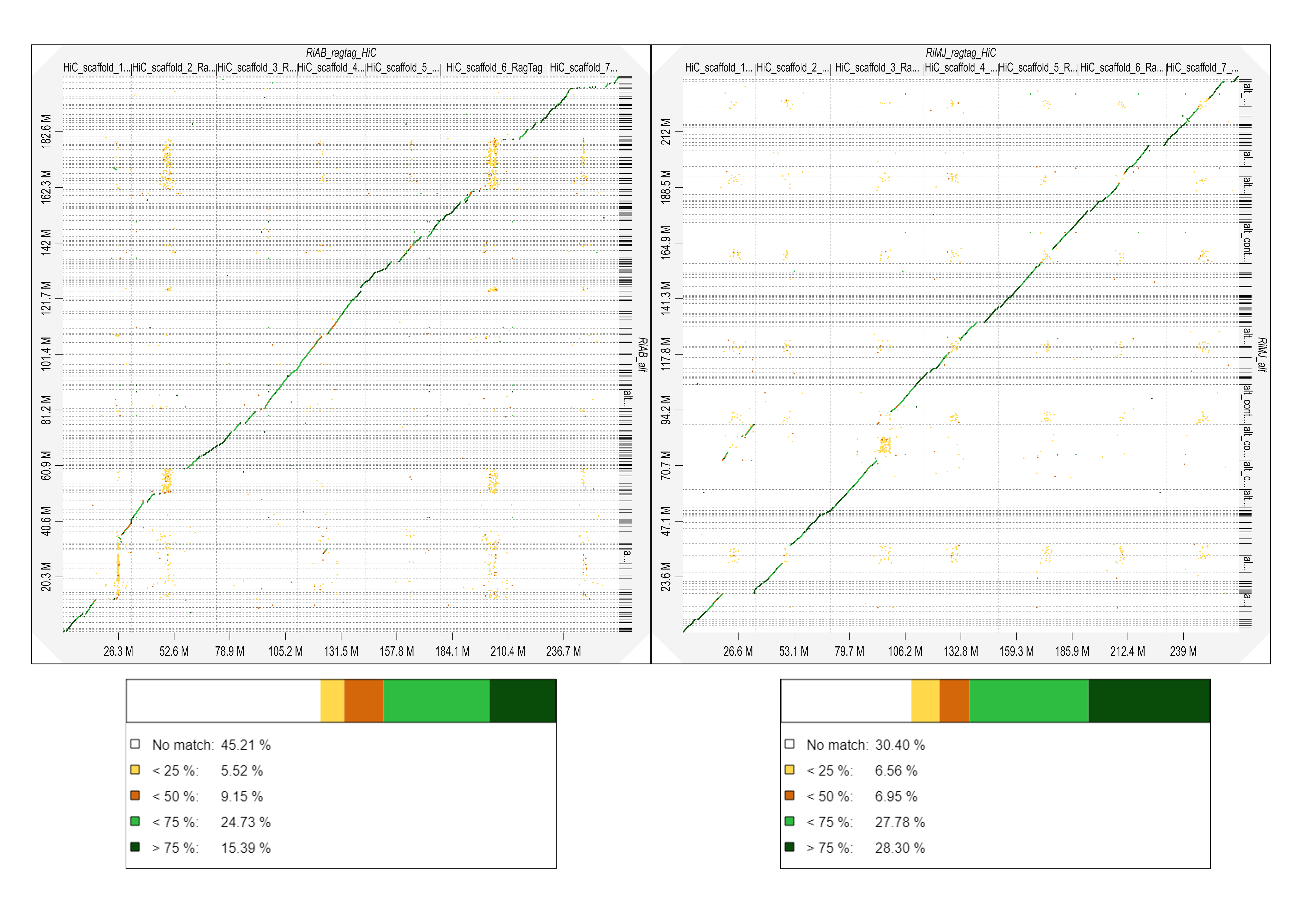

Supplement: S2 Fig — (PNG) [file pone.0285756.s002.png]
